# Impact of 1024‐Matrix Size on Perforating Artery Visualisation in Cerebral Computed Tomography Angiography Using a 64‐Slice CT Scanner

**DOI:** 10.1002/jmrs.70055

**Published:** 2026-03-17

**Authors:** Hokuto Nagumo, Yuki Nagata, Mayumi Maruko, Jun Sakai, Yusuke Fujiwara, Joma Oikawa

**Affiliations:** ^1^ Department of Radiology Sapporo Shuyukai Hospital Sapporo Japan; ^2^ Department of Neurosurgery Sapporo Shuyukai Hospital Sapporo Japan; ^3^ Department of Radiological Technology Japan Healthcare University Sapporo Japan

## Abstract

**Introduction:**

Computed tomography angiography (CTA) is essential for preoperative evaluation of intracranial vessels; however, the visualisation of small perforating arteries remains challenging with conventional systems. This study investigated whether increasing the reconstruction matrix size from 512 to 1024 pixels improves the visualisation of perforating arteries in cerebral CTA using a standard 64‐slice computed tomography (CT) scanner.

**Methods:**

The study comprised phantom and clinical components. Physical image properties were assessed through Task Transfer Function (TTF) and Noise Power Spectrum (NPS) analyses. In the clinical phase, 41 patients underwent cerebral CTA with both 512‐ and 1024‐matrix reconstructions. Quantitative analysis was performed to determine the peak CT values of the anterior choroidal artery and standard deviation in the basal cistern. Two independent observers (CT radiographers) evaluated the visualisation of anterior choroidal and posterior thalamoperforating arteries using a 5‐point scale.

**Results:**

Physical metrics showed minimal differences between matrix sizes, with 5% TTF values of 0.862 and 0.867 cycles/mm for 512 and 1024 pixels, respectively. The anterior choroidal artery exhibited higher peak CT values with 1024 pixels (139.31 ± 27.02 HU vs. 136.85 ± 26.69 HU, *p* < 0.001). The qualitative assessment revealed significant improvements in vessel visualisation with 1024 pixels, particularly for the posterior thalamoperforating artery, where high‐quality visualisation increased from 17.07% to 39.03%.

**Conclusions:**

Although increasing the matrix size from 512 to 1024 pixels did not significantly affect the physical image quality metrics, it enhanced the visual detection of small perforating arteries through enhanced sampling density. This optimization technique offers a practical approach for enhancing preoperative vessel evaluation using existing CT technology without requiring specialised equipment.

## Graphical abstract

This study investigated whether using a larger image matrix (1024 × 1024 pixels instead of 512 × 512) improves the visibility of tiny brain arteries on CT scans performed with standard 64‐slice scanners. While physical image quality measurements remained unchanged, the larger matrix provided finer digital sampling of blood vessel structures, resulting in significantly improved visualisation of small perforating arteries. This simple post‐processing technique can enhance preoperative vascular imaging without requiring specialised equipment or additional radiation exposure.

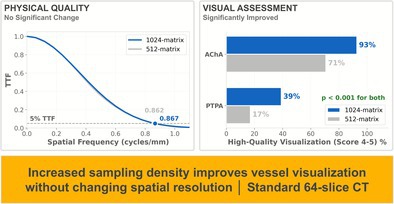

## 1 Introduction

Computed tomography angiography (CTA) is essential in preoperative planning, offering critical anatomical details that may not be fully captured using conventional angiography [1]. One key vessel of concern is the anterior choroidal artery (AChA), which must be carefully preserved during surgical clipping of internal carotid artery aneurysms. This artery has a diameter of approximately 0.7–1.2 mm [2] and supplies blood to several crucial brain structures [3]. However, the precise visualisation of such small intracranial arteries remains challenging with conventional CTA due to limitations in spatial resolution [4]. Among various parameters affecting computed tomography (CT) spatial resolution, the reconstruction matrix size represents one potential approach for improving the visualisation of these fine vascular structures.

Recent studies have investigated the impact of increasing the matrix size on image quality across various CT applications. In chest CT imaging, a matrix size of 1024 pixels exhibited superior image sharpness compared with conventional 512‐matrix reconstructions, although this technique is associated with increased image noise that could affect diagnostic confidence [5]. Similar advantages have been reported in CT colonography, where larger matrix sizes improved the detection of small lesions [6]. Although 64‐slice CTA has proven effective for detecting intracranial aneurysms [7] and ultra‐high‐resolution CT has shown promising results for visualising cerebral perforators [8], the potential benefits of increasing the matrix size on conventional energy integrating detector (EID) CT systems (standard 64‐slice scanners without ultra‐high‐resolution capability) for the visualisation of perforating arteries remain unexplored. This lack of research represents a significant disparity in the literature, particularly considering the widespread availability of conventional CT systems and their crucial role in routine neurosurgical planning.

Therefore, this study aimed to evaluate the impact of using a 1024‐pixel reconstruction matrix on the visualisation of perforating arteries in cerebral CTA performed with a conventional 64‐slice CT scanner. Phantom studies were conducted to assess fundamental image properties through the Task Transfer Function (TTF), that characterises spatial resolution performance, and Noise Power Spectrum (NPS), that quantifies noise characteristics. Subsequently, quantitative and qualitative evaluations were conducted on clinical images, with a particular focus on the AChA and posterior thalamoperforating artery (PTPA). This analysis aimed to determine whether increasing the matrix size enhances the detection and evaluation of these critical perforating vessels in daily clinical practice without requiring specialised CT equipment.

## 2 Materials and Methods

### 2.1 Study Design and Setting

This observational study was conducted at our institution between September 2022 and December 2023. The study comprised two parts: a phantom study to evaluate physical image properties and a clinical study to assess perforating artery visualisation. The Ethics Committee of Sapporo Shuyukai Hospital approved this study (approval number: 2023‐14), and the requirement for informed consent was waived due to the retrospective nature of the analysis.

### 2.2 Phantom Study

Physical assessment was performed using a quality assurance phantom (GE Healthcare, Chicago, IL, USA). Images were acquired using a 64‐slice CT scanner (Revolution Ascend, GE Healthcare) with the following parameters: tube voltage, 120 kV; tube current, 305 mA; rotation time, 0.5 s/rotation; beam width, 20 mm; helical pitch, 0.531; and scan field of view, 32 cm (Head).

The reconstruction parameters were as follows: display field of view, 20 cm; slice thickness, 0.625 mm; reconstruction interval, 0.625 mm; ASiR‐V, 0%; reconstruction kernel, Detail; and matrix sizes of both 512 and 1024 pixels. The Detail kernel emphasises high‐frequency components compared with the Standard kernel, thereby improving spatial resolution at the cost of increased image noise; this kernel was selected to maximise vessel edge definition in CTA applications. For the TTF analysis, 20 images were averaged for each measurement, and this process was repeated five times to obtain the final TTF values. The NPS was calculated from three sets of 100 images each, with the results averaged for the final analysis.

TTF and NPS analyses were conducted using ImageJ 1.52a software program (National Institutes of Health, Bethesda, MD, USA) and CT measure (version 0.98f; Japanese Society of CT Technology, Japan, http://www.jsct‐tech.org/) [9]. For the 1024‐matrix size images, the central 512 × 512‐pixel region was extracted using ImageJ before analysis because CT measure was designed to process images with matrix sizes up to 512. This extraction limits the TTF and NPS analysis to a portion of the full 1024‐matrix field of view; however, the central region encompasses the clinically relevant anatomy and provides a valid comparison of physical image properties between matrix sizes.

### 2.3 Clinical Study

#### 2.3.1 Patient Consent Statement

This retrospective study used anonymized data from patients who had provided comprehensive informed consent for the use of their medical information in research studies at Sapporo Shuyukai Hospital. The study was conducted at Sapporo Shuyukai Hospital. All personal identifiers were removed from the data before analysis to protect patient privacy and confidentiality in accordance with ethical guidelines.

#### 2.3.2 Patient Population

We retrospectively reviewed consecutive patients who underwent cerebral CTA at our institution. The final analysis included 41 patients (12 males and 29 females). The indications for CTA included cerebral aneurysms, brain tumours, and occlusive cerebrovascular disease. The exclusion criteria were as follows: (1) patients with severe image quality degradation due to motion artefacts; and (2) patients in whom the course of the AChA was affected by partial volume effects, beam hardening artefacts from bone, or mass lesions.

#### 2.3.3 CT Protocol

Clinical images were acquired using the same CT scanner with the following parameters: tube voltage, 120 kV; tube current modulated by automatic exposure control with noise index of 7.0; rotation time, 0.5 s/rotation; beam width, 20 mm; helical pitch, 0.531; and scan field of view, 32 cm (Head).

Images were reconstructed with a display field of view of 12 cm, slice thickness of 0.625 mm, reconstruction interval of 0.3125 mm, and Detail kernel. Matrix sizes of 512 and 1024 pixels were used for comparison. No iterative reconstruction (ASiR‐V: 0%) was applied.

Contrast medium was administered according to body surface area using the Livingston formula (980 mgI/m^2^/s). The injection duration was 15 s, and optimal scan timing was determined using a test bolus tracking method [10, 11].

#### 2.3.4 Quantitative Analysis

**FIGURE 1:**
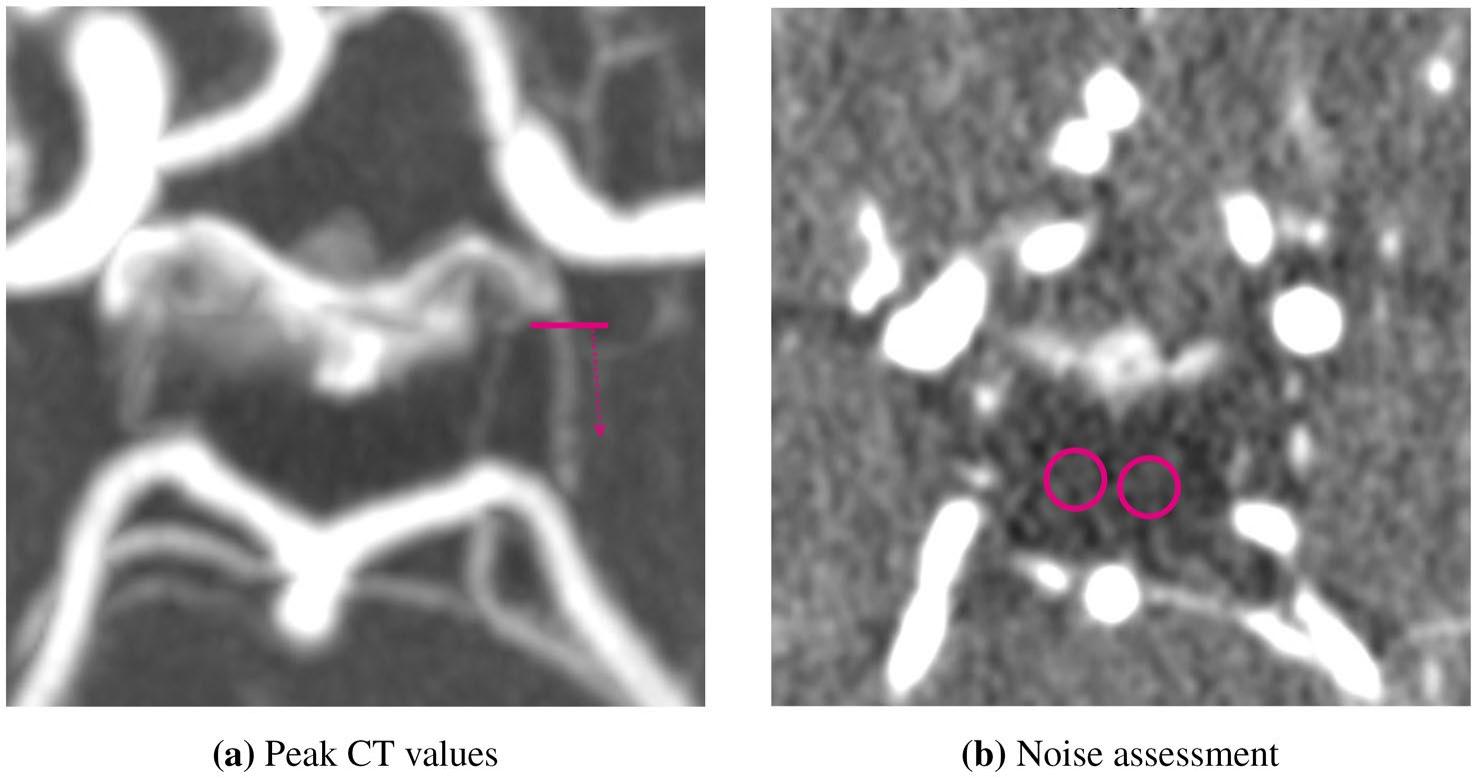
Quantitative assessment methods for CTA image analysis. (a) Measurement of peak CT values in the anterior choroidal artery (AChA) using profile curves. The red line indicates the measurement location along the vessel course, excluding the first 2 mm from the origin to avoid partial volume effects. (b) Noise evaluation method using standard deviation measurements in the basal cistern. Two circular regions of interest (ROIs) of 10 mm^2^ (red circles) were placed in homogeneous areas, and the average standard deviation was calculated.

A single investigator conducted all quantitative measurements. Peak CT values (in Hounsfield Units [HU]) of the AChA were assessed using 5‐mm slab maximum intensity projection (MIP) images (Figure 1a). Measurements were taken along the vessel path, omitting the initial 2 mm from the origin to minimise partial volume effects. At each point, profile curves were drawn perpendicular to the vessel axis to determine the maximum CT value.

For noise assessment, standard deviation (SD) values of the basal cistern were collected on 0.625‐mm source images. Two circular regions of interest (ROIs) of 10 mm^2^ were placed in homogeneous areas of the basal cistern, and the average of these two measurements was used for analysis (Figure 1b). Care was taken to avoid visible vessels and adjacent structures when placing the ROIs.

To maintain consistency when comparing the 512‐ and 1024‐matrix reconstructions, all measurements were performed at the same anatomical locations using an identical measurement method. Furthermore, uniform window settings were applied across all evaluations to reduce variability in visual interpretation.

#### 2.3.5 Qualitative Analysis

**FIGURE 2:**
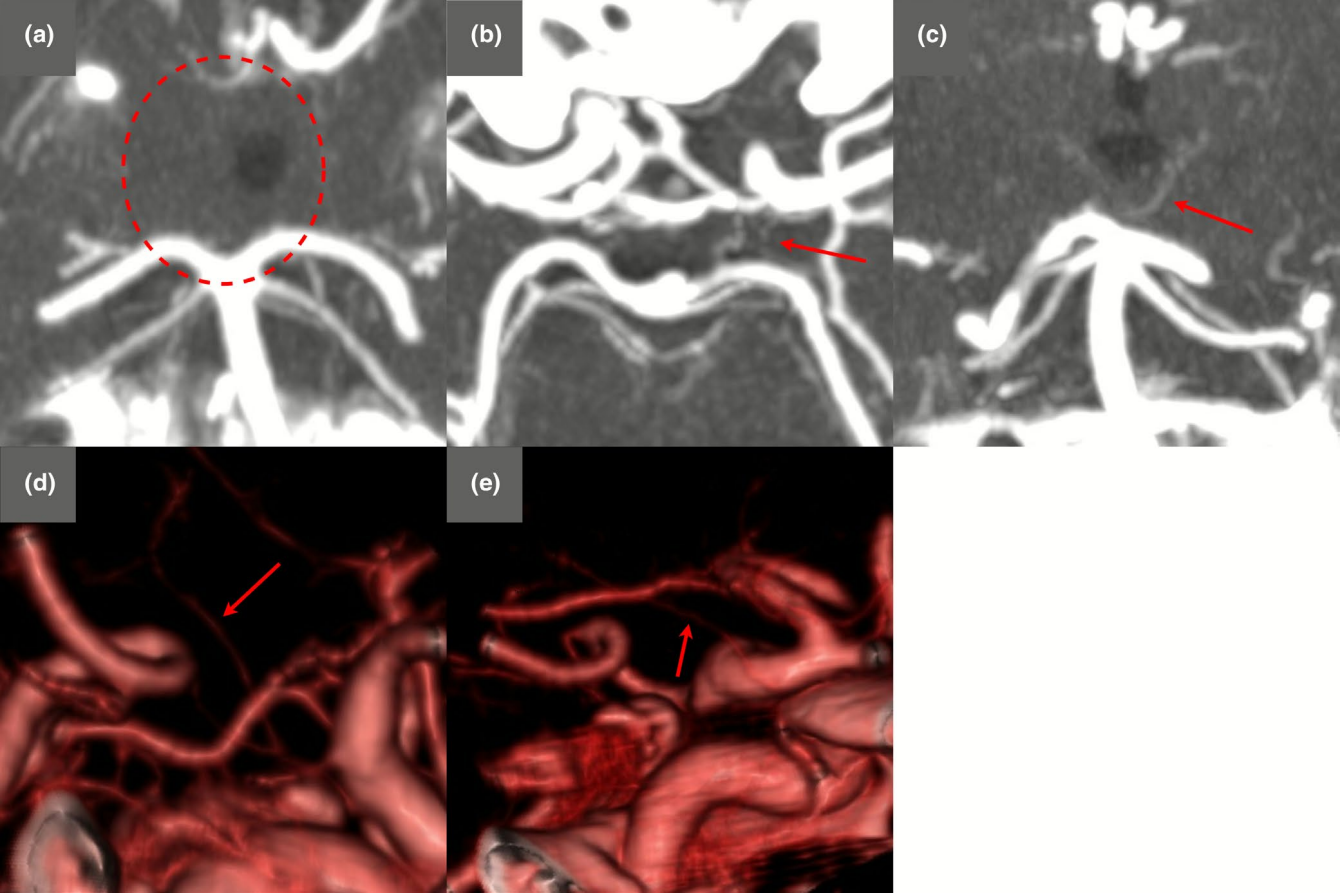
Representative images illustrating the 5‐point grading scale for perforating artery visualisation. (a) Score 1: Artery not identifiable on 10‐mm slab MIP (red dotted circle indicates the expected location of the vessel). (b) Score 2: Artery partially visible; however, it appears to be discontinuous on 10‐mm slab MIP (red arrow). (c) Score 3: Artery continuously visible from origin to periphery on 10‐mm slab MIP (red arrow). (d) Score 4: Artery clearly visible on 10‐mm slab MIP with confirmation possible on perforator‐specific volume rendering (VR) (red arrow). (e) Score 5: Artery clearly visible on 10‐mm slab MIP with confirmation possible on standard VR (red arrow). This 5‐point scale was used by two independent observers to evaluate anterior choroidal artery and posterior thalamoperforating artery visualisation in both 512 × 512 and 1024 × 1024 matrix reconstructions.

All image evaluations were performed using a specialised workstation (Ziostation2 Ver 2.9.8.5, Ziosoft Inc., Tokyo, Japan). Two independent CT radiographers with 4 and 12 years of experience in CT imaging, respectively, evaluated the visualisation quality of the AChA and PTPA. The observers were blinded to the matrix size and clinical information. The assessment used a 5‐point Likert‐type scale: 1 = not identifiable on 10‐mm slab MIP; 2 = partially visible but discontinuous on 10‐mm slab MIP; 3 = continuously visible from origin to periphery on 10‐mm slab MIP; 4 = clearly visible on 10‐mm slab MIP with confirmation possible on perforator‐specific volume rendering (VR); 5 = clearly visible on 10‐mm slab MIP with confirmation possible on standard VR (Figure 2). Slab MIP images were evaluated using a window width of 300 HU and window level of 100 HU. Perforator‐specific VR was displayed with a window level of 70 HU and sharpness of 80, whereas standard VR used a window level of 100 HU and sharpness of 80.

#### 2.3.6 Statistical Analysis

Statistical analyses were completed using Python (version 3.11.3). Before analysis, data normality was assessed using the Shapiro–Wilk test. For quantitative variables, such as peak CT values and SD measurements, paired *t*‐tests were applied because the data represented continuous variables obtained from the same subjects under two different conditions (512‐ and 1024‐matrix reconstructions).

For the qualitative analysis, interobserver agreement was assessed using weighted kappa statistics, which indicated substantial agreement (*κ* = 0.768, 95% confidence interval [CI]: 0.68–0.84). The Wilcoxon signed‐rank test was then used to compare visual evaluation scores because these represented ordinal data. The analysis included 82 observations (41 patients evaluated by two observers). *p*‐values < 0.05 were used to denote statistical significance.

## 3 Results

### 3.1 Phantom Study

**FIGURE 3:**
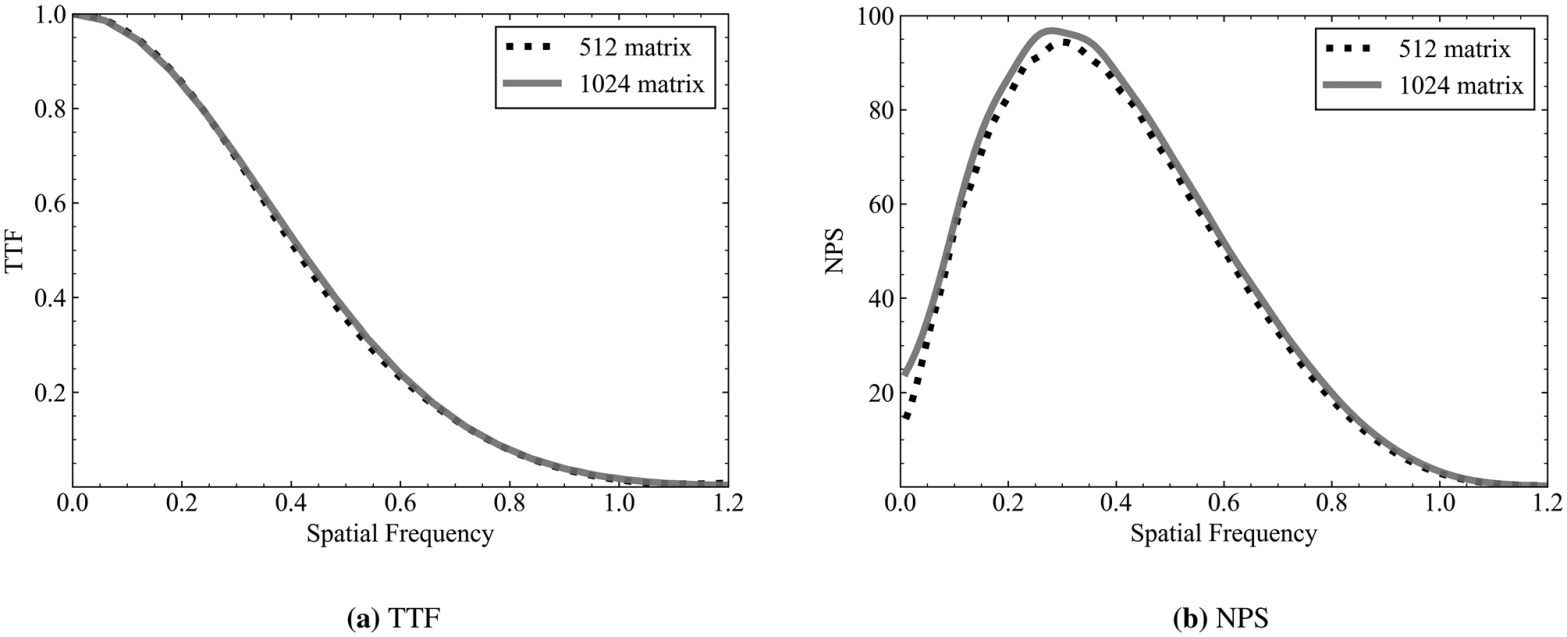
Results of (a) Task Transfer Function (TTF) and (b) Noise Power Spectrum (NPS) curves for 512‐matrix and 1024‐matrix sizes.

The 5% TTF values were similar between matrix sizes, with 0.862 cycles/mm for the 512‐matrix and 0.867 cycles/mm for the 1024‐matrix. The NPS measurements exhibited no substantial differences between the two matrix sizes, as demonstrated in Figure 3.

### 3.2 Clinical Study

#### 3.2.1 Patient Characteristics

The study included 41 patients (29 females and 12 males) with a mean age of 64.0 ± 14.5 years. The mean height and weight were 159.6 ± 7.5 cm and 59.9 ± 13.2 kg, respectively.

#### 3.2.2 Quantitative Analysis

Normal distribution was confirmed by the Shapiro–Wilk test for both peak CT values and standard deviation values (*p* < 0.05), justifying the use of paired *t*‐tests. The peak CT values of the AChA were significantly higher with the matrix size of 1024 pixels (139.31 ± 27.02 HU) than with the matrix size of 512 pixels (136.85 ± 26.69 HU) (*p* < 0.001); however, the absolute difference of approximately 2.5 HU is unlikely to be clinically meaningful. The SD values in the basal cistern exhibited no significant difference between the matrix sizes of 512 (9.07 ± 1.22 HU) and 1024 pixels (9.12 ± 1.23 HU) (*p* = 0.062).

#### 3.2.3 Qualitative Analysis

**FIGURE 4:**
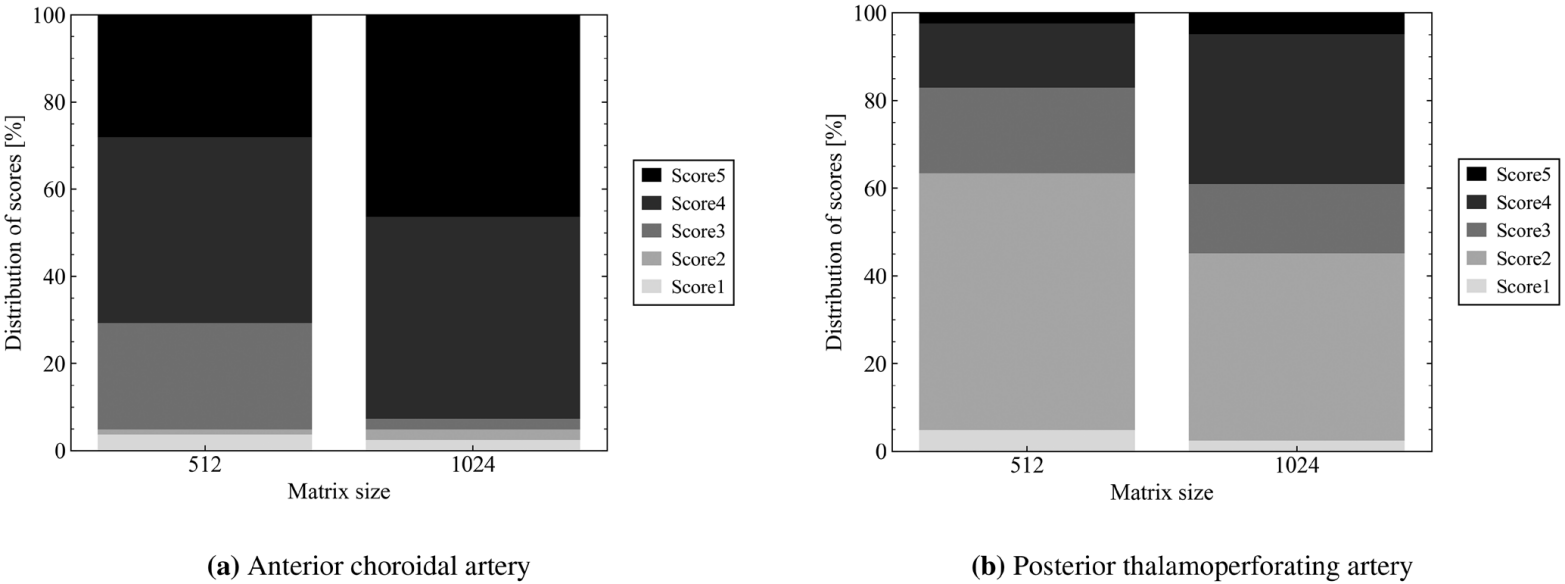
Distribution of visual evaluation scores shown in 100% stacked bar charts. (a) represents the distribution for the anterior choroidal artery, whereas (b) exhibits the distribution for the posterior thalamoperforating artery.

Interobserver agreement for the visual evaluation showed substantial agreement with a weighted kappa value of 0.768. The distribution of visual evaluation scores for both arteries is presented in Figure 4. The Wilcoxon signed‐rank test indicated a significant enhancement in vessel visualisation when using the 1024‐pixel matrix compared with the 512‐pixel matrix for both arteries evaluated (*p* < 0.001 for both).

For the AChA, the 1024‐matrix reconstruction resulted in higher visualisation scores, with the proportion of excellent visualisation (score 5) increasing from 28.05% with 512‐matrix reconstruction to 46.34% with 1024‐matrix reconstruction. Similarly, good visualisation (score 4) shifted from 42.68% to 46.34%. The proportion of poor visualisation (scores 1–2) remained unchanged at 4.88% for both matrix sizes.

The PTPA also exhibited significant improvement with the matrix size of 1024 pixels. The proportion of good‐to‐excellent visualisation (scores 4–5) increased from 17.07% with the matrix size of 512 pixels to 39.03% with the matrix size of 1024 pixels. Notably, the percentage of poor visualisation (score 2) decreased from 58.54% with the matrix size of 512 pixels to 42.68% with the matrix size of 1024 pixels.

## 4 Discussion

This study examined how matrix size influences the visualisation of perforating arteries in cerebral CTA using a conventional 64‐slice CT scanner. The results demonstrated two principal findings. First, physical image quality metrics—including TTF, NPS, and overall image noise—remained essentially unchanged between 512‐ and 1024‐matrix reconstructions. Although the 1024‐matrix showed statistically higher peak CT values in the AChA, the actual difference was minimal (approximately 2–3 HU) and is not considered clinically meaningful; this small magnitude is unlikely to influence diagnostic interpretation or vessel visualisation in practice. Second, qualitative assessment demonstrated improved visualisation of both the AChA and PTPA with the 1024‐matrix setting. The proportion of high‐quality scores (ratings of 4 or 5) increased considerably, particularly for the PTPA, which exhibited a 22% improvement in detection rates.

Our investigation revealed that increasing the reconstruction matrix from 512 to 1024 pixels had minimal impact on physical image quality metrics and CT values. The 5% TTF values were nearly identical (0.862 and 0.867 cycles/mm for the 512‐matrix and 1024‐matrix, respectively), and no substantial differences were observed in the NPS measurements. This finding differs from previous studies that showed improvements in TTF and changes in NPS with increased matrix sizes [6]. However, those studies employed nonlinear image reconstruction techniques that can alter image quality characteristics depending on the reconstruction conditions and object properties [12]. In contrast, our study employed filtered back projection to eliminate nonlinear reconstruction influences. Although previous clinical studies reported enhanced visualisation of fine structures with larger matrix sizes [13], we observed minimal changes in peak CT values. This can be attributed to the sampling theorem [14]. With a 12‐cm display field of view, the 512‐matrix achieved a pixel size of 0.234 mm, which was adequately small relative to the system's cutoff frequency of approximately 0.9 cycles/mm, as shown in our TTF analysis (Figure 3). This suggests that further reduction in pixel size would not contribute to improved vessel visualisation.

Despite minimal differences in physical measurements, qualitative assessment exhibited significant improvements in vessel visualisation with the matrix size of 1024 pixels. This apparent discrepancy between physical metrics and visual evaluation can be explained by the increased sampling density of small vessels at higher matrix sizes. The TTF and NPS metrics reflect intrinsic imaging system properties—primarily detector element size and reconstruction algorithm—rather than pixel dimensions; consequently, these physical metrics remained essentially unchanged despite the fourfold increase in pixel count. When reconstructing three‐dimensional vascular structures, however, the smaller voxel dimensions achieved with the matrix size of 1024 pixels allow for more precise digital sampling of vessel continuity [15]. Although increasing the sampling rate does not enhance the fundamental spatial resolution, as shown by our TTF analysis, it does provide more detailed spatial information regarding vessel structures [16]. This benefit is particularly pronounced for small perforating arteries, where maintaining vessel continuity is crucial for accurate detection and evaluation.

The practical relevance of our findings extends beyond theoretical image enhancement. Recent neurosurgical research has highlighted the vital need to preserve perforating arteries during various surgical interventions [17]. Surgical complications from perforating artery injury can lead to significant postoperative deficits [18], and careful evaluation of these vessels is crucial in aneurysm surgery to prevent ischemic events [19]. Our method for improving perforating artery visualisation by increasing the matrix size offers a practical solution that can be implemented on existing 64‐slice CT scanners, without requiring specialised ultra‐high‐resolution CT equipment or additional radiation exposure. Because this technique involves post‐processing rather than acquisition protocol changes, implementation may be straightforward; at our institution, cerebral CTA is routinely reconstructed at 512 × 512 pixels, with additional magnified reconstruction at 1024‐matrix applied to clinically critical regions. However, increasing the matrix size quadruples the pixel count and data volume, which may affect storage capacity, PACS and network traffic, and workstation CPU/GPU resources during post‐processing tasks such as MPR, MIP, and three‐dimensional rendering. The workflow impact of these considerations was not quantified in the present study. Furthermore, the present study assessed image quality improvements only; whether these enhancements translate into measurable changes in diagnostic accuracy or surgical decision‐making remains to be determined in future outcome‐focused investigations.

This study has several limitations that should be acknowledged. First, this was a single‐centre study using a single‐vendor 64‐slice CT system with filtered back projection reconstruction; the results may not be directly generalizable to other CT platforms, vendors, or reconstruction algorithms, including iterative and deep learning‐based methods. Second, the sample size of 41 patients is moderate, and larger multicenter studies involving diverse patient populations and CT systems would be valuable to validate and extend these findings. Third, although we demonstrated improved visualisation of perforating arteries, the clinical impact of this improvement—such as changes in diagnostic accuracy, surgical planning decisions, or patient outcomes—was not assessed. Fourth, workflow parameters including reconstruction time, data transfer duration, and post‐processing workload were not measured; these factors may influence the practical feasibility of routine 1024‐matrix implementation. Future studies should investigate whether enhanced vessel visualisation translates into improved surgical planning and reduced complications, ideally with prospective designs that capture clinical endpoints. Future research could also explore the potential benefits of combining increased matrix size with other optimization techniques, such as advanced reconstruction algorithms or contrast injection protocols, to enhance the visualisation of small intracranial vessels.

## 5 Conclusions

This study evaluated the effect of increasing the reconstruction matrix size on the visualisation of perforating arteries in cerebral CTA using a conventional 64‐slice CT scanner. Although the shift from a 512‐ to a 1024‐matrix did not significantly affect physical image quality metrics or CT attenuation values, it resulted in a clear improvement in the visual detection and evaluation of small perforating arteries. The enhanced visualisation was achieved through increased sampling density of vascular structures, even though overall spatial resolution and noise characteristics remained comparable. These improvements were particularly evident in the qualitative assessments of the AChA and PTPA, where the number of high‐quality visualisations significantly increased. Our findings suggest that implementing a 1024‐matrix size in cerebral CTA can serve as a simple and cost‐effective strategy to enhance preoperative vascular imaging without requiring specialised CT equipment. This optimization can be readily integrated into clinical settings, offering improved anatomical detail that supports more detailed surgical planning and potentially reduces procedure‐related risks, particularly in cases where precise evaluation of perforating arteries is crucial for successful surgical outcomes.

## Funding

The APC was funded by Japan Healthcare University.

## Ethics Statement

The study was conducted in accordance with the Declaration of Helsinki, and approved by the Ethics Committee of Sapporo Shuyukai Hospital (protocol code 2023–14 and date of approval 12 March 2024).

## Consent

This retrospective study used anonymized data from patients who had provided comprehensive informed consent for the use of their medical information in research studies at Sapporo Shuyukai Hospital. All personal identifiers were removed from the data before analysis to protect patient privacy and confidentiality in accordance with ethical guidelines.

## Conflicts of Interest

The authors declare no conflicts of interest.

## Data Availability

The data presented in this study are available on request from the corresponding author. The data are not publicly available due to ethical and data privacy regulations.
